# B7-1 and PlGF-1 are two possible new biomarkers to identify fracture-associated trauma patients at higher risk of developing complications: a cohort study

**DOI:** 10.1186/s12891-024-07789-0

**Published:** 2024-08-29

**Authors:** Regina Breinbauer, Michelle Mäling, Sabrina Ehnert, Gunnar Blumenstock, Tobias Schwarz, Johann Jazewitsch, Felix Erne, Marie K. Reumann, Mika F. Rollmann, Benedikt J. Braun, Tina Histing, Andreas K. Nüssler

**Affiliations:** 1 Siegfried-Weller-Institute, BG Unfallklinik Tuebingen, Eberhard Karls University Tuebingen, 72076 Tuebingen, Germany; 2 Department of Clinical Epidemiology and Applied Biometry, Eberhard Karls University Tuebingen, Silcherstrasse 5, 72076 Tuebingen, Germany; 3 Department of Traumatology and Reconstructive Surgery, BG Unfallklinik Tuebingen, Eberhard Karls University Tuebingen, Schnarrenbergstr. 95, 72076 Tuebingen, Germany

**Keywords:** Trauma, Fracture repair, B7-1, PlGF-1, Obesity, Alcohol consumption, Gender, Complication

## Abstract

**Background:**

Around 10% of fractures lead to complications. With increasing fracture incidences in recent years, this poses a serious burden on the healthcare system, with increasing costs for treatment. In the present study, we aimed to identify potential ‘new’ blood markers to predict the development of post-surgical complications in trauma patients following a fracture.

**Methods:**

A total of 292 trauma patients with a complete three-month follow-up were included in this cohort study. Blood samples were obtained from 244 of these patients. Two complication groups were distinguished based on the Clavien-Dindo (CD) classification: CD grade I and CD grade III groups were compared to the controls (CD 0). The Mann-Whitney U test was used to compare the complication groups to the control group.

**Results:**

Analysis of the patients’ data revealed that risk factors are dependent on sex. Both, males and females who developed a CD III complication showed elevated blood levels of B7-1 (*p* = 0.015 and *p* = 0.018, respectively) and PlGF-1 (*p* = 0.009 and *p* = 0.031, respectively), with B7-1 demonstrating greater sensitivity (B7-1: 0.706 (male) and 0.692 (female), PlGF-1: 0.647 (male) and 0.615 (female)). Further analysis of the questionnaires and medical data revealed the importance of additional risk factors. For males (CD 0: 133; CD I: 12; CD III: 18 patients) alcohol consumption was significantly increased for CD I and CD III compared to control with *p* = 0.009 and *p* = 0.007, respectively. For females (CD 0: 107; CD I: 10; CD III: 12 patients) a significantly increased average BMI [kg/m^2^] from 25.5 to 29.7 with CD III was observed, as well as an elevation from one to three comorbidities (*p* = 0.003).

**Conclusions:**

These two potential new blood markers hold promise for predicting complication development in trauma patients. Nevertheless, further studies are necessary to evaluate the diagnostic utility of B7-1 and PlGF-1 in predicting complications in trauma patients and consider sex differences before their possible use as routine clinical screening tools.

## Introduction

In recent years, there has been an increase in the incidence of fractures [1]. In Germany, the fracture incidence increased by 14% between 2009 and 2019 [2]. Fracture incidences vary between different countries depending on type of fracture, age and sex [3]. Considering that approximately 10% of these fractures result in complications, the total number of complications is also on the rise [4]. These complications can range from delayed wound healing and infections to non-union or, in severe cases, even fatalities [5]. This poses a significant burden on the healthcare system, often necessitating additional treatments such as surgeries, and it can also have detrimental effects on long-term patients outcome [6].

Markers are often evaluated in elderly patients, as well as in the presence of a certain type of fracture or a health event requiring surgery [7]. In a standard blood draw, elevated levels such as C-reactive protein (CRP), albumin or hemoglobin can be used to assess the risk of infection [8]. In contrast, an increase in immune cells such as leukocytes is considered an appropriate response after a fracture and promotes fracture healing [9, 10]. Therefore, conventional blood markers appear to have limited suitability for use in trauma patients to predict potential complications.

Several risk factors that increase the likelihood of complications are often associated with the fracture itself. Factors like the severity and type of fracture, as well as the necessity of surgical intervention, are beyond healthcare professionals’ control and contribute to the overall outcome [5, 11, 12]. However, recent research has highlighted the substantial influence of a patient’s pre-existing health condition on fracture healing [6]. Comorbidities such as diabetes, rheumatoid arthritis, osteoporosis, medication, smoking, obesity, and others [13, 14] can impact the immune system, directly or indirectly affecting the healing process. It is important to note that the impact of these risk factors may vary depending on the type and localization of the fracture.

In recent years, the importance of gender has increasingly become the focus of medical studies regarding the progression of certain diseases, their treatment strategies and prevention [15, 16]. This results in different health risks for males and females [6] as hormone balance, immune system, symptoms of disease, and response to pharmaceuticals are different depending on the sex of the patients [17]. In addition, the gender-specific shape of the pelvis and weight distribution has a major impact on the musculoskeletal system - with implications for mobilization and convalescence after trauma surgery.

Although we can’t change a patient’s health status and gender before a fracture occurs, there is potential value in identifying individuals at higher risk of developing complications after a fracture. This information could lead to more tailored treatment approaches aimed at reducing or even preventing complications.

Fracture healing is a complex process with different, overlapping phases, during which, different cell types and cytokines interact and regulate each other. Due to the disruption of the surrounding tissue, a hematoma forms around the fracture site and the immune system gets activated. The first phase of fracture healing is called the inflammatory phase. In addition to macrophages and neutrophils from the innate immune system, B- and T- T-cells from the adaptive immune system are necessary to regulate the inflammatory response [18]. In this first stage of fracture healing, several key cytokines such as interleukin (IL) 1 and 6, Transforming growth factor- β (TGF-β), bone morphogenetic proteins (BMPs), and more play important roles. Furthermore, due to a disruption of the blood flow at the fracture site, the revascularization process is an important part of early fracture healing. Mesenchymal stem cells (MSCs) are attracted to the fracture site and are responsible for the formation of a collagen network. This process takes about two weeks after which a soft callus is formed. In the second phase, the repair phase, which takes several months, more matrix proteins are expressed. Through the formed blood vessels, more cells migrate and differentiate into osteoblasts and osteoclasts allowing a hard callus bone formation. In the final phase, the bone is remodeled to regenerate normal bone structure. This takes several months to years [19–23].

There are different ways to document the postoperative course. Categories can be formed by dividing into minor and major complications, or adverse and severe adverse events. Such terms are subjective and can change from study to study. The present study uses the Clavien-Dindo (CD) classification as it is based on the type of therapy that is needed after the initial surgery and can be applied more universally throughout the cases [24, 25].

Since conventional blood markers have shown limited utility in predicting potential complications in trauma patients at the time of hospital admission, the aim of this study is to identify potential “new/alternative” blood markers that could predict the development of postoperative complications in trauma patients. These blood markers can be assessed during a standard blood draw and could be an elegant, modern supplement to risk management. In addition, questionnaires and medical data were used to identify additional risk factors in this cohort study. The occurrence or non-occurrence of post-surgical complications was set as an endpoint.

## Methods

### Ethic-code

The study was approved by the Ethics Committee according to the Declaration of Helsinki (1964) and its most recent amendment. Patients were interviewed and clinically relevant data were collected according to the ethical approval number 346/2015/BO2, amended on March 30, 2020, and accepted by the Ethics Committee of the Medical Faculty of the University Hospital and the Eberhard Karls University of Tübingen on July 9, 2020. The blood samples were taken as part of a standard (study-independent) blood sample collection procedure. All study participants signed a written consent form.

### Patient recruitment

Patients who were treated at a trauma level 1 center in Germany and underwent surgery after trauma, were included in this cohort study. Inclusion criteria were written consent, age between 18 and 69 years, inpatient hospitalization, not cognitively impaired, and being able to speak the local language. Consequently, exclusion criteria were patients under the age of 18 years or over the age of 69 years, no complete 3 months follow-up, conservative therapy, polytrauma and cognitively impaired patients (e.g., Dementia or otherwise not able to read and fill out the questionnaires). Patient data, such as medical history, were collected from the patient’s medical file. In addition, patients were interviewed using different questionnaires: Finnish Diabetes Risk Score (FINDRISK) [26], Alcohol Use Disorders Identification Test (AUDIT-C) [27], and Smoking-ultra-short [28]. Three months after surgery the perioperative recovery was investigated through follow-ups. During these follow-ups, patients were interviewed again, and their recovery was independently assessed. Adverse events were documented by the treating physician and classified according to CD.

### Blood collection

One EDTA- and one Serum- a vial of 4 mL each were collected as early as possible at the patient’s admission, during a standard blood withdrawal in the clinic. The samples were stored on ice and processed after 30 to 60 min by centrifuging for 10 min at 4 °C and 1,000 g. Serum and EDTA samples were aliquoted and stored at -80 °C until later processing.

### Group assignment according to CD classification

Complications during reconvalescence were classified according to the CD classification and split into three distinct patient groups. The control group comprised patients with no complications (CD 0). We differentiated two complication groups: one for patients with CD grade I (CD I) and another for patients with CD grade III (CD III). Notably, there were no CD grade II, IV, or V cases documented in our study cohort. CD grades are summarized in Table 1.

**Table 1:** Clavien-Dindo classification

Grade	Definition
Grade I	Any deviation from the normal postoperative course without the need for pharmacological treatment or surgical, endoscopic, and radiological interventions
Grade II	Requiring pharmacological treatment with drugs other than such allowed for grade I complications. Blood transfusions and total parenteral nutrition are also included
Grade III	Requiring surgical, endoscopic, or radiological intervention
Grade IV	Life-threatening complication requiring IC/ICU-management
Grade V	Death of patient

IC: intermediate care; ICU: intensive-care-unit

### Identification of risk factors for the best possible matching of serum samples

The patients’ data were analyzed using JMP 16.2 software (SAS Institute Inc., Cary, NC, USA), to identify potential risk factors based on the questionnaire responses. A partitioning analysis was conducted to distinguish between the control group (with no complications) and the two complication groups. Based on the identified risk factors, a meticulous best possible person-to-person matching process was performed to minimize the influence of any other variables that could potentially contribute to complications. For this, the primary aspects of gender, age, drinking, or BMI should be similar between the complication groups and the controls. This matching process was used for the screening with different cytokine arrays to identify markers that do not rely on the pre-existing conditions of the patients. In the following ELISA analysis all patients, regardless of their risk factors were analyzed to assess the overall capacity of the identified markers.

### Cytokine arrays

Human Cytokine Antibody Array C5 and Human Immune Checkpoint Array C1 were chosen to screen different factors affecting the immune system. Human Angiogenesis Antibody Array C2 and Human TGF beta Array C2 were selected to screen angiogenic factors. All arrays (produced from RayBiotech, distributed by BioCat, Heidelberg, Germany) were performed following the manufacturer’s protocol. Serum samples from each group were pooled together as one sample and incubated overnight on the membranes. Chemiluminescent signals were recorded with an INTAS Chemocam, CCD Camera. The images were quantified with ImageJ (NIH, Bethesda, MA, USA) after subtracting the background. Signal intensities were normalized to the positive controls. All samples were measured in duplicate.

### Enzyme-linked-immunosorbent-assay

Levels of eight different targets (Table 2) were determined in the patient’s serum or EDTA samples by Enzyme-Linked-Immunosorbent-Assay (ELISA) according to the manufacturer’s instructions. Samples were diluted and the concentration was determined using a standard curve, respectively. All samples were measured in duplicate, and the standards were measured in triplicate.

**Table 2:** List of performed ELISA

Target	Sample	Order No	Company	Dilution factor
hmr BMP-2	Serum	900-K255	Peprotech	2
h IL-1β	Serum	900-K95	Peprotech	2
h PlGF-1	Serum	900-K307	Peprotech	2
h TGF-β2	Serum	DY302	RnD Systems	5
h CTLA-4	Serum	DY386-05	RnD Systems	10
h TIM-1	EDTA	DY1750B	RnD Systems	/
h B7-1	EDTA	DY140	RnD Systems	1.25
h B7-2	EDTA	DY141-05	RnD Systems	1.25

h: human; m: murine; r: rat; BMP-2: Bone morphogenetic protein 2; IL-1β: Interleukin 1β; PlGF-1: placenta growth factor 1; TGF-β2: Transforming growth factor 2; CTLA-4: Cytotoxic-T-Lymphocyte associated molecule 4; TIM-1: T-cell immunoglobulin Mucin 1; B7-1: Cluster of differentiation 80; B7-2: Cluster of differentiation 86

### Statistics

For statistical analysis, Graph Pad Prism 8 (San Diego, CA, USA), Microsoft Office (Microsoft Corporation, Redmond, USA), and JMP 16.2 (SAS Institute Inc., Cary, NC, USA) were used. Partitioning, predictor screening and hierarchical cluster platforms were used as analyzing tools in the JMP software. Normality assumption was checked graphically using histograms and analytically by means of parameters skewness and kurtosis for symmetry and peakedness of the data, respectively. Normally distributed data are presented as mean and standard deviation, while non-normally distributed data are presented as median and interquartile range (IQR). Categorical data are reported as numbers and percentages. An a priori minimum sample size calculation was performed using the www.powerandsamplesize.com platform, where power analysis showed that in an unbalanced sample design large effect sizes can be detected in the terminology of Cohen (1988) [29] Assuming that approximately 10% of the patients will develop a complication, the sampling ratio κ (N_A_ / N_B_) was set at 9. Further assuming a standard deviation (σ) of a maximum of 4 times the mean of the controls, a minimum sample size of *N* = 160 (N_A_ = 144 controls and N_B_ = 16 complications) was identified to identify changes ≥ 4-fold at a power (1 – β) of 80% and a significance level (α) of 0.05. Non-parametric two-way ANOVA (The Mann-Whitney U test) was used to identify changes between groups, when the development of complication in both sexes was investigated. Data are shown as box plots (Box and Whiskers – Median; 5–95%) or in heat maps (mean). Significance level (α) was defined as 0.05. The resulting probabilities (* *p* < 0.05, ** *p* < 0.01, *** *p* < 0.001, and **** *p* < 0.0001) are indicated in corresponding figures.

## Results

### Patient recruitment and cohort description

**Fig. 1:**
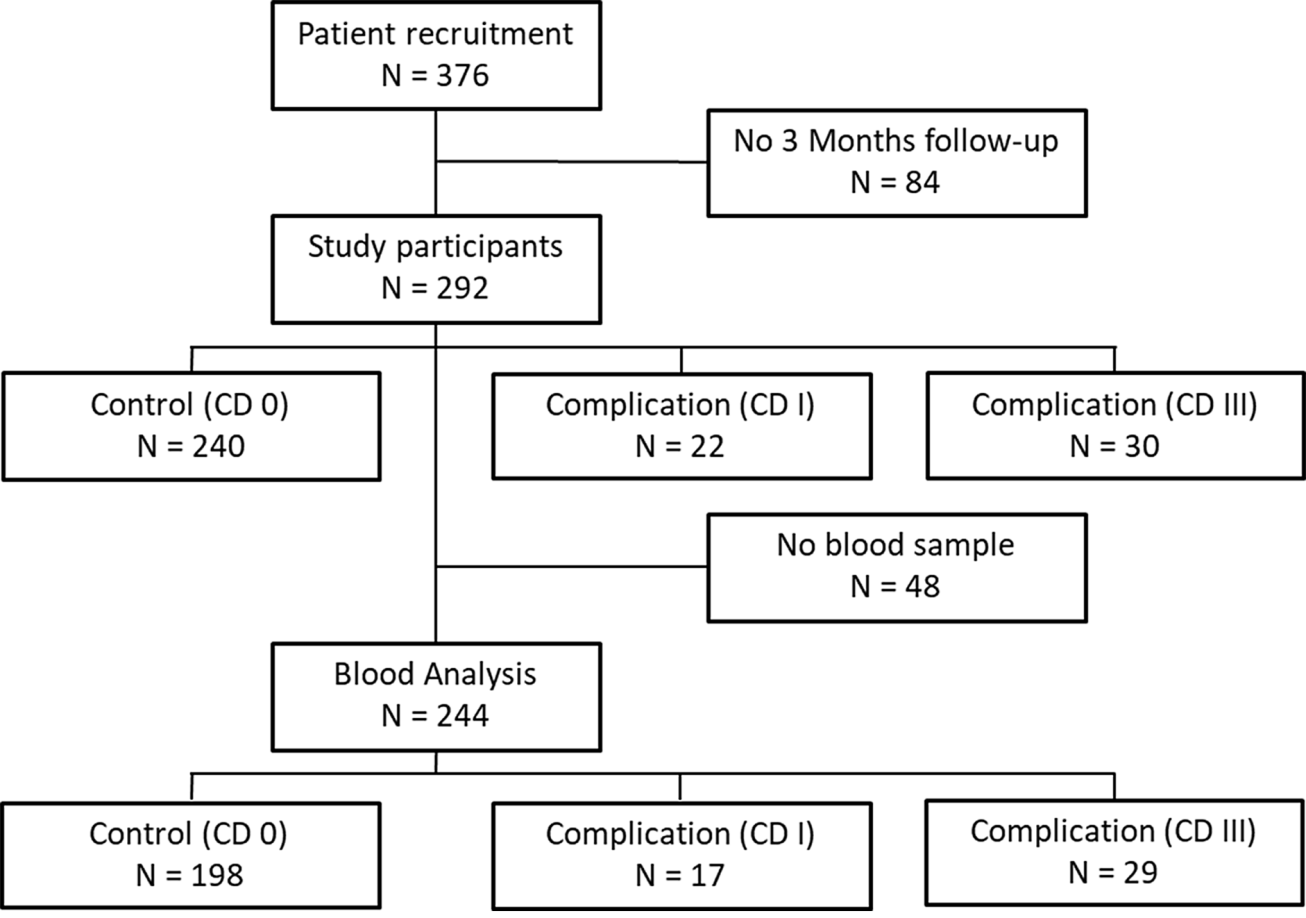
Patient flow chart

This study enrolled a total of 376 trauma patients, of whom 292 had a fully documented three-month follow-up. These patients were categorized into different groups based on their CD classification, which included the control group (CD 0), complication group I (CD I), and complication group III (CD III). Additionally, 48 patients were excluded from the molecular biological analysis because of missing blood samples, as indicated in Fig. 1.

Initially, we characterized the three groups: CD 0, CD I, and CD III, and the change from the two complication groups to the control was investigated. The gender distribution remained consistent across these groups, with a slightly higher representation of males (56%) overall. The average age of all patients was 48.6 ± 14.8 years, and there were no significant age differences between the control group and the two groups with complications.

As the severity of complications increased, we observed a corresponding rise in the average length of hospital stay (LOS). Specifically, LOS increased only slightly from 7 to 7.5 days for CD I patients (*p* = 0.326) and to 10 days for CD III patients (*p* = 0.077).

The AUDIT-C Score displayed a noticeable increase from CD 0 to CD III (*p* = 0.062), and the patient-reported frequency of alcohol consumption showed a significant difference, with CD III patients reporting a higher frequency compared to the control group (*p* = 0.021).

Similarly, the FINDRISK Score was slightly elevated for the complication groups, but the most significant difference emerged when examining BMI. BMI was found to be significantly increased from 25.9 ± 5.5 (CD 0) to 28.1 ± 5.9 for CD III patients (*p* = 0.026). Although calculated pack-years (1 Pack-year defined as 20 cigarettes per day for one year) showed increased median levels with complications, the groups were not significantly different from each other.

Furthermore, even though the median values for comorbidities stayed at one for patients with CD0 and CDIII their significance was at *p* = 0.052. For a summary, please refer to Table 3. Other variables such as fracture location, which showed no influence on the complication rate in this study, are not presented.

**Table 3:** Baseline characteristics of the study population

	CD 0	CD I	CD III	All patients	*p*-Value (to CD 0)
Age [years]	47.9 ± 15.2 (18–69)	52.4 ± 11.4 (31–69)	50.7 ± 13.5 (21–69)	48.6 ± 14.8 (18–69)	0.278
Sex (m/f)	133/107 (55%/45%)	12/10 (55%/45%)	18/12 (60%/40%	163/129 (56%/44%)	0.699
Length of stay [days]	7.0 (3.0–13.0) (1–44)	7.5 (4.0 -14.5)(2–34)	10.0 (4.8–15.3) (1–31)	7.0 (3.0–13.0) (1–44)	0.077
BMI [kg/m^2^]	25.9 ± 5.5 (16.5–53.1)	26.6 ± 5.2 (16.5–41.4)	28.1 ± 5.9 (29.5–48.5)	26.1 ± 5.6 (16.5–53.1)	0.026
FINDRISK [score]	7.0 (4.0–10.0)(0–20)	8.0 (4.0–12.3)(1–18)	7.5 (4.8–12.3)(1–21)	7.0 (4.0–10.0)(0–21)	0.204
Alcohol frequency [Point score]	2.0 (1.0–3.0)(0–4)	3.0 (0.0–3.0)(0–4)	2.0 (1.8–3.0)(0–4)	2.0 (1.0–3.0) (0–4)	0.021
AUDIT-C [score]	2.0 (1.0–4.0) (0–11)	3.0 (0.0–5.0) (0–8)	3.0 (2.0–4.0)(0–7)	3.0 (1.0–4.0) (0–11)	0.062
Pack-years	0.5 (0.0–14.0) (0–145)	3.8 (0.0–10.0) (0–31)	4.5 (0.0–11.8) (0–50)	1.0 (0.0–13.2) (0–145)	0.734
Comorbidities [Number]	1.0 (0.0–2.0) (0–6)	0.5 (0.0–2.0) (0–2)	1.0 (0.0–2.3) (0–8)	1.0 (0.0–2.0) (0–8)	0.052

Age and BMI are shown as average ± standard deviation (range of values); sex is shown as number (male/female) and percentage; all other variables are shown as median (IQR) and (range of values); p-Values were calculated with Mann-Whitney Test to CD 0

### Patient data suggest gender differences in risk factors for complications

**Fig. 2:**
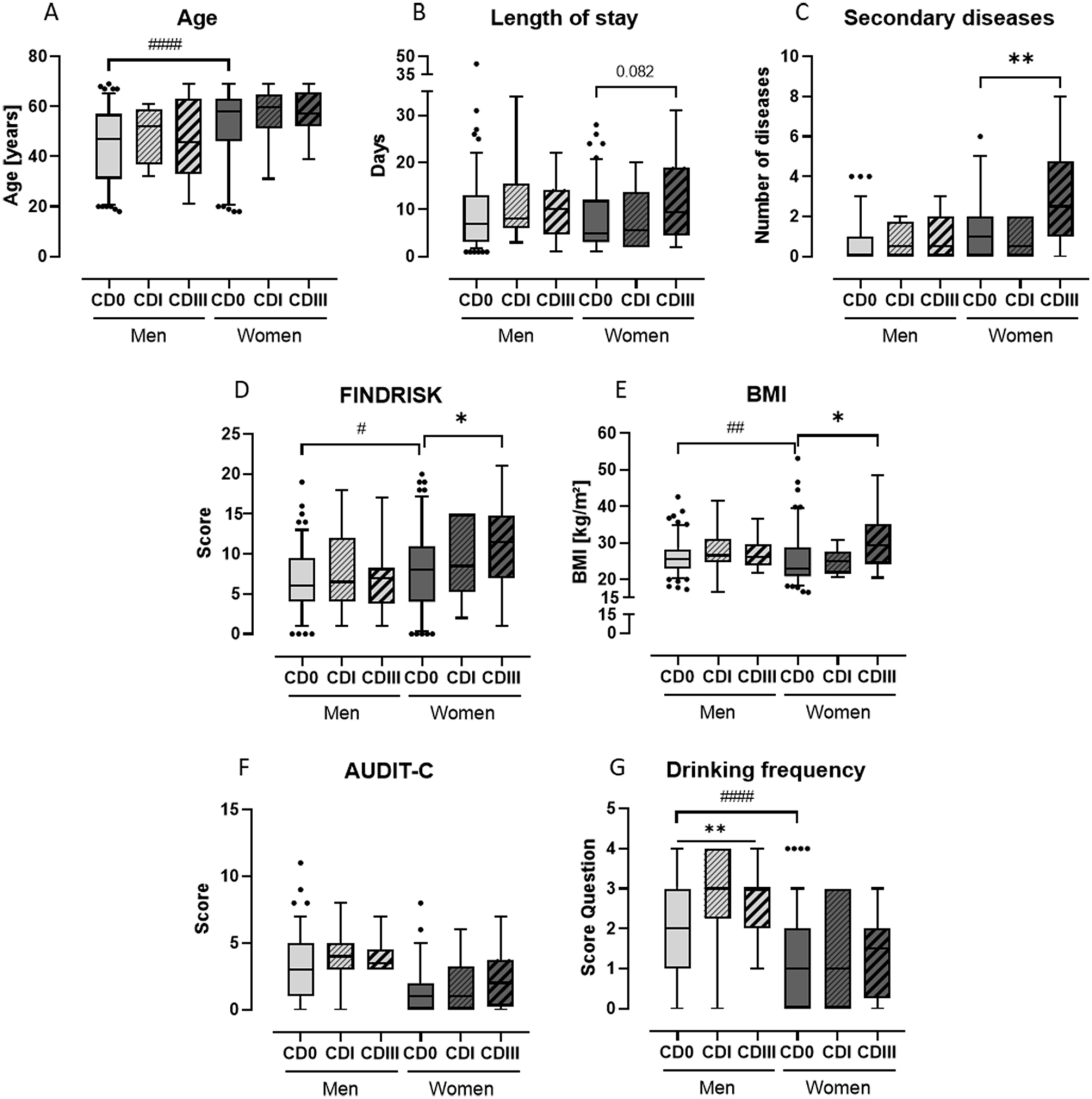
Gender-specific analysis of patient-related risk factors. Comparative analysis was done to the gender-specific control group (CD 0) as well as between CD 0 groups. Number of patients: Males: CD 0 = 133; CD I = 12; CD III = 18. Females: CD 0 = 107; CD I = 10; CD III = 12. */# *p* < 0.05; ## *p* < 0.01; #### *p* < 0.0001. Data are shown as box-plots with 5–95 percentile

The analysis of questionnaires and patient data has revealed gender-specific variations in risk factors for complications, as illustrated in Fig. 2. While age did not significantly differ between the two complication groups and the controls, it was noteworthy that females in the control were, on average, 8 years older than males with CD 0 (*p* < 0.0001), see Fig. 2A. Additionally, the average LOS was2 days longer for males with CD 0 compared to females with CD 0 (*p* = 0.091). An increase in the LOS was also observed for females with CD III (*p* = 0.082), see Fig. 2B. This observation extended to comorbidities, with female patients showing an elevation in comorbidities from one (CD 0) to three (CD III) with *p* = 0.003, see Fig. 2C.

Results from the questionnaires further highlighted gender-dependent differences. Both the FINDRISK score (Fig. 2D) and calculated BMI (Fig. 2E) were higher for females with CD III (*p* = 0.028 and *p* = 0.038, respectively) compared to the control group. Notably, the control groups exhibited significant gender differences (*p* < 0.0001), with males generally having higher BMI but lower FINDRISK scores (*p* = 0.030).

The analysis also revealed an increased level in the AUDIT-C score and drinking frequency among males with CD I and CD III complications compared to CD 0. While the AUDIT-C score only pointed out any differences (CD I: *p* = 0.188 and CD III: *p* = 0.248), see Fig. 2F, the comparison reached statistical significance when assessing the frequency of alcohol consumption (CD I: *p* = 0.009 and CD III: *p* = 0.008), see Fig. 2G. In the control groups, males with CD 0 were already consuming alcohol significantly more frequently than females (*p* < 0.0001).

### Cytokine arrays

The sample size for CD I patients was below the calculated minimum size of 10 patients per group in both males (*N* = 9) and females (*N* = 8) and they were therefore not included in further blood analysis.

**Fig. 3:**
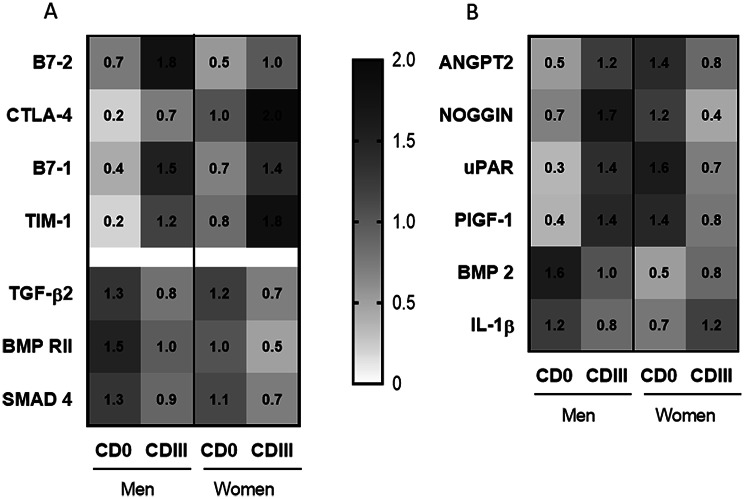
Relative expression values of selected cytokines from cytokine arrays. Data are normalized to the overall mean of each target. B7-2: Cluster of differentiation 86; CTLA-4: Cytotoxic T-Lymphocyte associated molecule 4; B7-1: Cluster of differentiation 80; TIM-1: T-cell immunoglobulin Mucin 1; TGF-β2: Transforming growth factor 2; BMP RII: Bone morphogenetic protein receptor II; SMAD 4: SMAD family member 4; ANGPT2: Angiopoietin 2; uPAR: urokinase receptor; PlGF-1: Placenta growth factor 1; BMP-2: Bone morphogenetic protein 2; IL-1β: Interleukin 1β

To explore and identify potential novel blood markers for predicting complications, we conducted four different cytokine arrays (refer to the Methods section) using pooled serum samples from patients. A comprehensive screening was performed, covering a total of 151 different factors. Only factors meeting the criteria of having a relative expression exceeding 10% of the positive control and displaying a difference of more than 40% between the control and complication groups in both males and females are presented in Fig. 3. An overview of the number of excluded cytokines for each array is shown in Table 4. Notably, we conducted gender-specific analyses of serum levels.

It is worth highlighting that all proteins exhibiting elevated expression levels in the complication groups are involved in the regulation of the immune response [30]. Conversely, specific proteins, including TGF-β2, BMP RII, and SMAD 4, which are integral components of the TGF-β pathway [31], showed reduced expression in the complication groups (Fig. 3A).

Of particular interest, our analysis revealed seven cytokines with contrasting expression trends between males and females in the complication group compared to controls (Fig. 3B). Additionally, we observed notable differences in the expression levels within the control groups (CD 0) between males and females.

**Table 4:** Overview of excluded cytokines per array

	C5	IC	ANG	TGFB	Total
< 10% of positive Control	37	16	13	2	**68**
< 40% between CD0 and CDIII	42	3	7	18	**70**
Excluded/Total measured cytokine	79/80	19/23	20/23	20/25	**138/151**

C5: Human Cytokine Antibody Array C5; IC: Human Immune Checkpoint Array C1; ANG: Human Angiogenesis Antibody Array C2; TGFB: Human TGF beta Array C2

### ELISA analysis

**Fig. 4:**
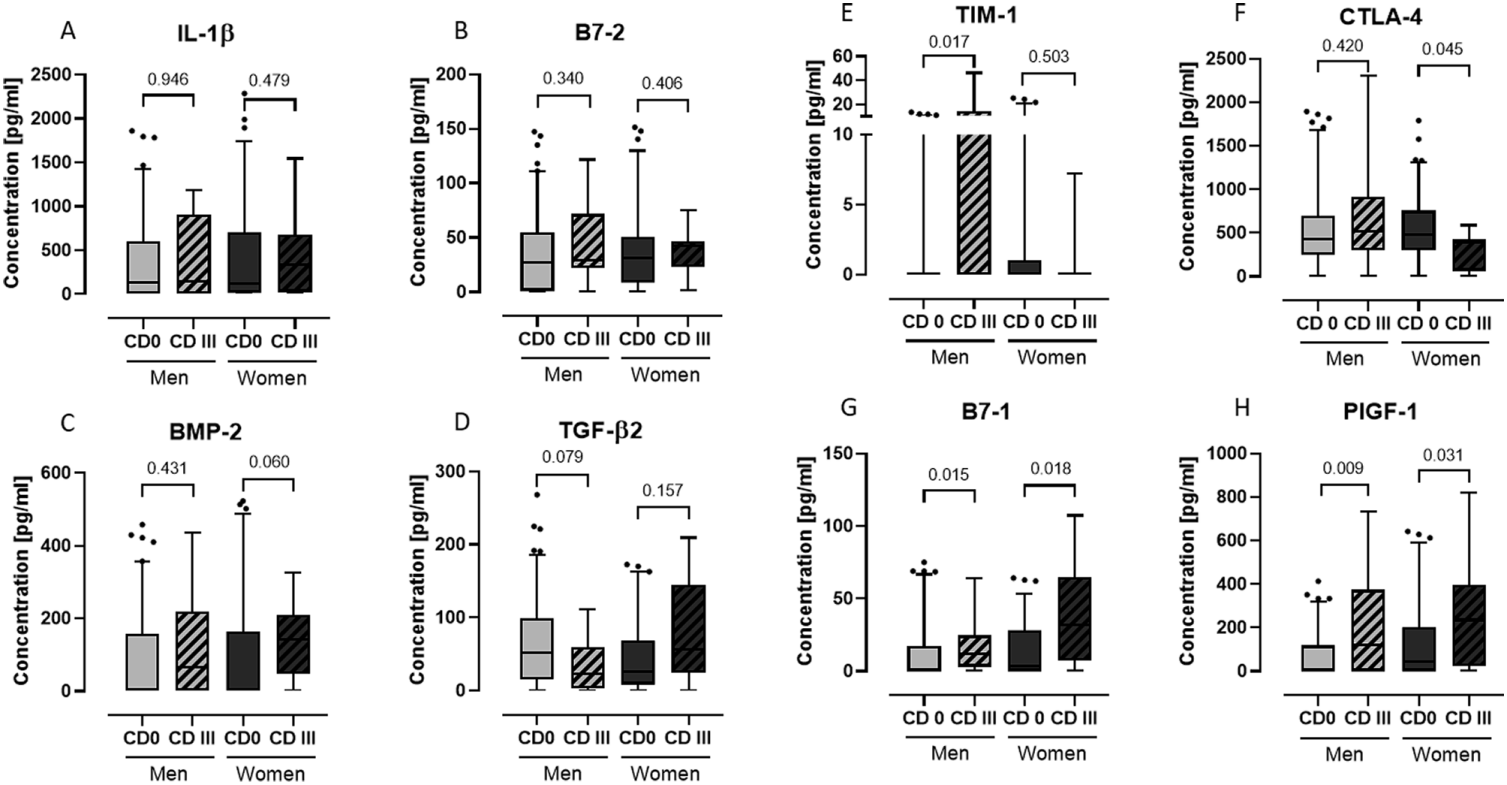
Comparative quantitative analysis of proteins by ELISA. Data are shown as box-plots with 5–95 percentile. Comparisons were performed to the gender-specific controls (CD 0) and Outliers were identified with ROUT 0.1%. Males: CD 0 = 109; CD III 17. Females: CD 0 = 86; CD III = 12. Technical replicates *n* = 2

To validate and quantify the results obtained from the cytokine array, we performed ELISA tests for each patient individually. However, it is important to note that not all results from the previous cytokine array screening were confirmed, as shown in Fig. 4.

For IL-1β and B7-2, the measured blood levels did not differ in the controls between males and females (*p* = 0.693 and *p* = 0.903, respectively). While IL-1β levels remained unchanged for male patients, the median IL-1β level in the CD III female group was 2.7 times higher than in the CD 0 female group (see Fig. 4A). Although we did not observe statistically significant differences between control and complication groups, there was a noticeable shift towards higher measured levels in B7-2 in CD III patients, while CD 0 patients displayed a wider range of blood levels (see Fig. 4B).

The controls for BMP-2 showed no differences (*p* = 0.835) with a median of approximately 5 pg/mL. BMP-2 levels exhibited a marked increase in females with CD III, reaching a median of 142 pg/mL (*p* = 0.060). The elevation of BMP-2 levels for males was less pronounced, at 65 pg/mL (*p* = 0.431), see Fig. 4C.

Circulating TGF-β2 levels between males and females in CD 0 were strongly different (*p* = 0.083). For males with CD III, TGF-β2 levels were lower by 50% (*p* = 0.079), whereas females with CD III had values 50% higher (*p* = 0.157), see Fig. 4D.

For the two targets, contrary gender-specific differences were observed, despite similar CD 0 levels (TIM-1: *p* = 0.302 and CTLA-4: *p* = 0.484) among the genders. TIM-1 levels were near the detection limit of the ELISA, yet males with CD III had significantly higher blood levels (*p* = 0.017), see Fig. 4E. In contrast, CTLA-4 was significantly lower (*p* = 0.045) for females with CD III compared to CD 0. Males, on the other hand, showed comparable levels with or without complications (*p* = 0.420), see Fig. 4F.

Uniform results were obtained for B7-1 and PlGF-1 blood levels. Both targets exhibited significantly increased levels in the complication groups. Interestingly, compared to males, PlGF-1 levels were significantly higher for females with CD 0 (*p* = 0.033), and there was a noticeable effect towards higher levels in B7-1 for females with CD 0 (*p* = 0.130) compared to males CD 0. In the complication groups, the median B7-1 level for females was 2.5 times higher than those in males, see Fig. 4G. Similarly, the median PlGF-1 level for females with CD III was twice as high as in males with CD III. Males showed a more pronounced, significant difference in PlGF-1 levels (*p* = 0.009) than females (*p* = 0.031), see Fig. 4H.

**Fig. 5:**
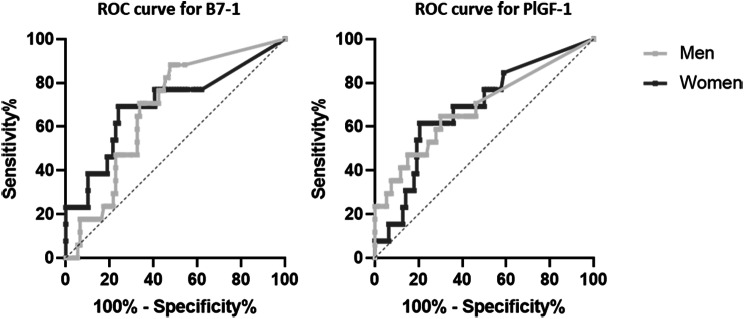
Receiver-operating-characteristics curve for B7-1 and PlGF-1 between CD 0 and CD III patients

The potential of B7-1 and PlGF-1 as biomarkers was further evaluated using receiver-operating characteristic (ROC) analysis, as depicted in Fig. 5. Statistical data are summarized in Table 5. It is noteworthy that the threshold values for males for B7-1 and PlGF-1 were lower than those for the corresponding female group. For B7-1, the Area under the Curve (AUC), Youden Index, and Likelihood Ratio were higher for females. Only the sensitivity was slightly better for males, while the specificity, at 0.760 for females, was 10% higher than that for males at 0.663.

When considering PlGF-1, a higher sensitivity of 0.647 was achieved for male patients, while females exhibited a higher specificity, with 0.795 compared to 0.700 for males. This observation is also reflected in the Youden Index and Likelihood Ratio, both of which were higher in females for both markers.

**Table 5:** Diagnostic accuracy for B7-1 and PlGF-1 in detecting CD III complication

Marker	Gender	AUC	Threshold [pg/mL]	Sensitivity	Specificity	Youden -Index	Positive Likelihood Ratio (LR+)
B7-1	Male	0.679	> 9.5	0.706	0.663	0.369	2.1
	Female	0.700	> 29	0.692	0.760	0.452	2.9
PlGF-1	Male	0.686	> 55	0.647	0.699	0.346	2.1
	Female	0.682	> 210	0.615	0.795	0.410	3.0

Diagnostic accuracy for B7-1 and PlGF-1 in detecting CD III complication in males and females

## Discussion

In the present study, we aimed to identify potential ‘new’ blood markers to predict the development of post-surgical complications in trauma patients following a fracture. Analysis of the study cohort highlighted the importance of distinguishing between different types of complications. Specifically, the CD I group had comparable baseline characteristics to the controls. CD I complications are defined as ‘any deviation from the normal postoperative course’ [25] and typically do not require additional, invasive treatment. In contrast, the treatment of CD III complications was considerably more invasive, often involving revision surgeries. It is worth noting that surgical procedures, in general, come with inherent risks of complications [32] and result in high healthcare costs [33, 34]. Consequently, our study focused primarily on CD III complications for further analysis.

Moreover, our analysis underscored the importance of gender differentiation within the study group. Age and gender distribution mirrored the patterns observed in society. Males generally exhibit a higher risk of fractures at younger ages, often due to lifestyle factors [35]. Conversely, for females, the fracture risk increases with age, primarily due to osteoporotic changes [36]. In our study, females were, on average, 8 years older than males. Since geriatric patients (those aged 70 years and older) were excluded from our study, we achieved an evenly distributed gender representation.

Several studies have identified various comorbidities that are associated with an increased complication rate [20, 37, 38]. Roche et al. also demonstrated that the presence of three or more comorbidities, regardless of the specific comorbidities, significantly increased the complication rate, especially among elderly patients [39]. In our trauma patient cohort, this observation was evident among females with complications, where the average number of comorbidities increased to three. In contrast, males in our study were generally younger, with an average count of secondary diseases remaining below one. This gender difference in comorbidities can be attributed to the fact that secondary diseases tend to increase with age [40, 41].

LOS is traditionally analyzed in relation to increased healthcare costs [42] or in comparison to in-hospital complications before, during, or after surgery [43, 44]. However, in our study, we used LOS as a predictor for post-hospitalization complications. Interestingly, Marfil-Garza et al. previously set prolonged LOS in correlation with comorbidities, male gender, and increased mortality [45]. As females with complications in our study cohort had an average of three comorbidities (*p* = 0.003), their LOS was also, on average, 4 days longer than that of the control group. Furthermore, the overall longer LOS for males, observed in our study, aligns with these findings.

In addition to basic patient data, we employed supplementary questionnaires to identify further potential risk factors. Although smoking is often associated with an increased infection rate and impaired wound healing [46, 47], we did not observe a relationship between increased pack-years (PY) and CD I or CD III patients. There was only a minor increase in PY among CD III females, from 8 to 11 PY, which falls below the threshold for heavy smokers.

The FINDRISK Score assesses the risk of developing type 2 diabetes over the next 10 years, with scores above 11 points indicating an increased risk for diabetes [48, 49]. One of the primary contributors to the development of type 2 diabetes is obesity, typically defined as a BMI over 30 kg/m² [50]. Obesity and type 2 diabetes are known to be associated with an elevated fracture risk and negative effects on fracture healing [51–54]. When examining the distribution of BMI between males and females, our study cohort closely mirrored the distribution in German society [55, 56]. Data from 2017 indicated that the cumulative percentage of overweight and obese males was 61.6%, with an average BMI of 26.7 kg/m², compared to 46.7% and an average of 25.1 kg/m² for females. In our study, the BMI for CD 0 males and females was nearly identical at 26.2 and 25.5 kg/m², respectively. While males with complications did not exhibit any change in BMI, females with CD III displayed higher levels of obesity (mean BMI = 29.7 kg/m²), suggesting a potential impact on fracture healing. Similarly, the FINDRISK score, akin to BMI, indicated an increased risk of diabetes for females with CD III (mean score = 11 points), while average scores of males remained consistent across the groups. When comparing the BMI and the FINDRISK score for their suitability in screening patients with a higher risk of complications, the BMI emerged as favorable, as it is already documented in the patient files, requires no additional surveys, and has fewer influencing factors.

We assessed possible alcohol abuse using the AUDIT-C score [57], with a score of ≥ 4 for males or ≥ 3 for females indicating potential alcohol misuse [58]. An increase in complication rates for males with a score of ≥ 5 was noted by Bradley et al. [59]. In our study, a noticeable increase in scores was observed for males, from 3 points in the control group to 4 points with CD I and CD III complications, indicating a higher risk of developing complications. However, females consistently scored below 3 points across all groups, suggesting no risk of alcohol abuse and no influence on the post-operative outcome. Notably, when examining drinking frequency, one of the questions on the AUDIT-C, a significant increase was observed, from 2 to 4 times a month (2 points) in the control group to 2–3 times a week (3 points) for males with CD I and CD III complications. This finding underscores the importance of this question and the significance of considering it alongside the final AUDIT-C score, a pattern consistent with findings by Rubinsky et al., who found that alcohol intake of 2 drinks a day was a more accurate predictor of post-surgical outcomes than the AUDIT-C score alone [60]. This underscores the importance of individual questions within the AUDIT-C questionnaire and emphasizes the need to consider them alongside the final AUDIT-C score, as observed in this study.

The synopsis of the patient data indicate that many factors are interconnected, making it challenging to consider them individually. For instance, the younger age of males may explain why increased alcohol intake was the only factor identified as increasing the risk of complications. Conversely, for females, age played a role, and patient-related data revealed comorbidities, particularly obesity, which correlated with extended hospital stays and increased the risk of post-operative complications.

In our study, with a three-month follow-up period to document any complications, we specifically assessed factors related to early fracture healing. Analysis of the different cytokine arrays revealed 13 cytokines that met the criteria of relative expression compared to the positive control and exhibited differences between the control and complication groups for both males and females. As the primary focus of this study was to identify a blood marker that can be assessed during a standard blood draw, 5 targets were excluded from further analysis.

Among these targets, TNF-α, IL-6, and IL-1β are key inflammatory cytokines that play pivotal roles in modulating cell proliferation during fracture repair [61]. These pro-inflammatory factors are crucial for bone formation and are upregulated after a fracture. However, stress can also induce increased IL-1β levels [62], and continuously elevated IL-1β levels are observed in chronic inflammatory diseases [63]. While we found higher levels in CD III females, we cannot make definitive statements about their progression over time or whether excessively high IL-1β levels impair osteoblast formation. Existing research suggests that the absence of IL-1β may limit bone formation [61] which was not observed in these trauma patients.

Furthermore, the TGF-β superfamily, encompassing TGF-β isoforms, BMPs, and growth and differentiation factors (GDFs), plays a role in recruiting immune cells, mesenchymal stem cells (MSCs), and collagen formation [64, 65]. Different members of this superfamily exhibit varying peaks in expression during fracture healing, with substantial interactions and modulation among them, resulting in overlapping expression patterns over time [66]. Both TGF-β and BMP levels are utilized to assess improved fracture healing after treatment [67, 68]. Chaverri et al. demonstrated that lower levels of TGF-β1/β2 were found in non-union patients after 12 months [69], while no initial differences were evident. Although male patients with complications already displayed lower levels of TGF-β2 at the beginning of fracture healing, this was not observed for female patients. Additionally, the differences did not reach statistical significance. Similarly, while slightly higher levels of BMP-2 were detected in both complication groups, statistical significance was not achieved. These elevated BMP-2 levels could also be attributed to the timing of the blood sample collection after the fracture, as BMP-2 activation occurs at various phases of fracture healing, resulting in changing levels in the blood [70].

In the initial stage of fracture healing, various immune cells play a critical role in mediating inflammation [9]. This study delves into the analysis of several factors that regulate T-cell activation during this process.

TIM-1, also known as kidney injury molecule-1 (KIM-1), is typically associated with various diseases, including kidney ischemia, asthma, and different carcinomas [71, 72] and showed higher levels in patients who suffered hemorrhagic shock after injury [73]. It plays a vital role in T-cell-mediated immune responses by regulating immune cells [74, 75]. However, the role of TIM-1 in fracture healing has yet to be explored. In this study, higher levels of TIM-1 were detected in males with complications, whereas it was nearly undetectable in the controls. A study by Du et al. demonstrated that inhibiting TIM-1 can reduce tissue damage caused by inflammatory reactions, although this was investigated in the context of tumor formation [76]. The expression of TIM-1 following a fracture could potentially contribute to prolonged inflammation, thus impairing wound healing.

B7-1 (CD80), B7-2 (CD86), and CTLA-4 (CD152) belong to the same co-stimulatory factor family for T-cells. B7-1/2 are expressed on antigen-presenting cells (APCs) and can bind to CD28 and CTLA-4, which are proteins expressed on T-cells. This interaction is well-characterized for modulating T-cell function in response to inflammation and pathogens [30, 77]. While the binding of B7-1/2 to CD28 promotes T-cell proliferation and activity, CTLA-4 serves as a counterpart that limits T-cell function [78, 79]. Although T-cell activation is crucial for a successful immune response [80], a study by Nolan et al. revealed that CD80-knockout mice were less prone to sepsis due to reduced inflammation, underscoring the role of B7-1 as a dominant receptor in regulating the immune response [77]. It is noteworthy that T-cell activation during fracture healing is typically described through IL-17 A [9] rather than by the binding of B7-1/2 to CD28. In this study, although B7-2 showed unchanged blood levels, B7-1 was significantly higher in both complication groups compared to the controls. Additionally, CTLA-4 levels were decreased in females with CD III complications. CTLA-4 not only functions as a counterbalance to limit T-cell activation but is also expressed on mesenchymal stem cells (MSCs), where it plays a role in the anti-inflammatory properties of MSCs and their potential to differentiate into osteocytes [81].

Bozec et al. demonstrated that inhibiting CTLA-4 resulted in increased osteoclastogenesis (formation of bone-resorbing cells) [82]. Low osteocytes and increased osteoclast formation both negatively affect bone remodeling after a fracture. Further studies are needed to investigate whether this pathway of B7-1/2 activation of T cells could play a role in the development of complications.

PlGF-1, which plays a role in angiogenesis after fracture, has also been shown to be a distinct marker. PlGF-1, a homolog of VEGF, is a pro-angiogenic factor that stimulates blood vessel growth [83]. An animal study by Maes et al. showed that knockout of PlGF-1 resulted in non-union [84]. In contrast, Burska identified high PlGF-1 serum levels days after a fracture as a marker for non-union in humans. In this study, high PlGF-1 levels were also associated with impaired fracture healing.

### Limitations

One of the limitations that should be considered is the timing of blood sampling. The proteins analyzed in this study exhibit their peak expression in the days following the fracture, and they also react to the surgical intervention at the fracture site. Each day following the fracture can lead to variations in blood protein levels. For proteins that showed no significant changes (TGF-β2, BMP-2, IL-1β), this variability in timing may be one of the primary influencing factors to account for. Furthermore, although the group sizes met the calculated minimum sample size, further validation with an increased sample pool would help to validate the results. Other factors, such as the type of surgical intervention, were not controlled but could impact the development of complications.

## Conclusion

In this study, we identified two potential new markers, B7-1 and PlGF-1, that hold promise for predicting future complication development in trauma patients. B7-1, with its higher sensitivity for both sexes compared to PlGF-1, appears to be a more favorable candidate for use as a future screening method as it can detect more patients who may later develop complications. Moreover, our study revealed gender differences in cytokine profiles to identify patients at risk in trauma patients. Nevertheless, it is important to emphasize that further studies are needed to evaluate the effectiveness of B7-1 and PlGF-1 in predicting complications in trauma patients and consider gender differences before their possible use as routine clinical screening tools.

## Data Availability

No datasets were generated or analysed during the current study.
